# Right Ventricular Reverse Remodeling After Tricuspid Valve Surgery for Significant Tricuspid Regurgitation

**DOI:** 10.1016/j.shj.2022.100101

**Published:** 2022-11-04

**Authors:** Xavier Galloo, Maria Chiara Meucci, Jan Stassen, Marlieke F. Dietz, Edgard A. Prihadi, Pieter van der Bijl, Nina Ajmone Marsan, Jerry Braun, Jeroen J. Bax, Victoria Delgado

**Affiliations:** a Department of Cardiology, Leiden University Medical Center, Leiden, The Netherlands; b Department of Cardiology, Vrije Universiteit Brussel (VUB), Universitair Ziekenhuis Brussel (UZ Brussel), Brussels, Belgium; c Department of Cardiovascular and Thoracic Sciences, Catholic University of the Sacred Heart, Rome, Italy; d Department of Cardiology, Jessa Hospital, Hasselt, Belgium; e Hartcentrum, Ziekenhuis Netwerk Antwerpen (ZNA) Middelheim, Antwerp, Belgium; f Department of Cardio-Thoracic Surgery, Leiden University Medical Center, Leiden, The Netherlands; g Heart Center, University of Turku and Turku University Hospital, Turku, Finland

**Keywords:** Prognosis, Right ventricular remodeling, Tricuspid regurgitation, Tricuspid valve surgery

## Abstract

**Background:**

Changes in right ventricular (RV) dimensions and function after tricuspid valve (TV) surgery and their association with long-term outcomes remain largely unexplored. The current study evaluated RV reverse remodeling, based on changes in RV dimensions and function, after TV surgery for significant (moderate or severe) tricuspid regurgitation (TR) and their association with outcome.

**Methods:**

A total of 121 patients (mean age 63 ± 12 years, 47% males) with significant TR treated with TV surgery were included in this analysis. The population was stratified by tertiles of percentage reduction of RV end-systolic area (RVESA) and absolute change of RV fractional area change (RVFAC). Five-year mortality rates were compared across the tertiles of RV remodeling and independent associates of mortality were investigated.

**Results:**

Tertile 3 consisted of patients presenting with a reduction in RVESA ≥17.2% and an improvement in RVFAC ≥2.3% after TV surgery. Cumulative survival rates were significantly better in patients within tertile 3 of RVESA reduction: 90% vs. 49% for tertile 1 and 69% for tertile 2 (log-rank *p* = 0.002) and within tertile 3 of RVFAC improvement: 87% vs. 57% for tertile 1 and 65% for tertile 2 (log-rank *p* = 0.02). Tertiles 3 of RVESA reduction and RVFAC improvement were both independently associated with better survival after TV surgery compared to tertiles 1 (hazard ratio: 0.221 [95% CI: 0.074-0.658] and 0.327 [95% CI: 0.118-0.907], respectively).

**Conclusions:**

The extent of RV reverse remodeling, based on reduction in RVESA and improvement in RVFAC, was associated with better survival at 5-year follow-up of TV surgery for significant TR.

## Highlights

• Severe tricuspid regurgitation leads to right ventricular dilation and dysfunction.
• Right ventricular adverse remodeling and dysfunction are associated with survival.
• The association with survival after tricuspid valve surgery is largely unexplored.
• Patients presented significant reverse remodeling after tricuspid valve surgery.
• The magnitude of reverse remodeling was significantly associated with survival.

## Introduction

Significant (moderate or severe) tricuspid regurgitation (TR) is associated with poor survival, independent of left ventricular systolic function and pulmonary hypertension.^1^^,^^2^ Significant TR, when left untreated, may remain asymptomatic for a long time, despite inducing progressive right atrial and right ventricular (RV) dilation, dysfunction and finally, RV failure.^3^^,^^4^ Moreover, RV adverse remodeling together with RV dysfunction, and not only TR severity, are independently associated with survival in patients with medically treated significant TR.^3^, ^4^, ^5^, ^6^ Current guidelines for the management of valvular heart disease advise tricuspid valve (TV) surgery as a concomitant procedure to left-sided valve surgery in patients with severe TR or at an earlier phase if coexistent tricuspid annular dilatation (≥40 mm or ≥21 mm/m^2^) is present.^7^^,^^8^ Isolated TV surgery is recommended in symptomatic patients with severe primary or secondary TR in the absence of severe right- or left ventricular dysfunction or severe pulmonary hypertension.^7^^,^^8^

Left-sided reverse remodeling and the effect on left ventricular function after mitral valve surgery has been extensively studied.^9^^,^^10^ However, studies evaluating the changes in RV dimensions and function after TV surgery have shown inconsistent results.^6^^,^^11^, ^12^, ^13^, ^14^, ^15^, ^16^, ^17^, ^18^ Moreover, the association between RV reverse remodeling after TV surgery with survival remains largely unexplored. The aim of the current study was to (i) evaluate right-sided reverse remodeling after TV surgery and (ii) investigate the prognostic implications of RV reverse remodeling in patients undergoing TV surgery for significant TR.

## Material and Methods

### Study Population

**Figure 1:**
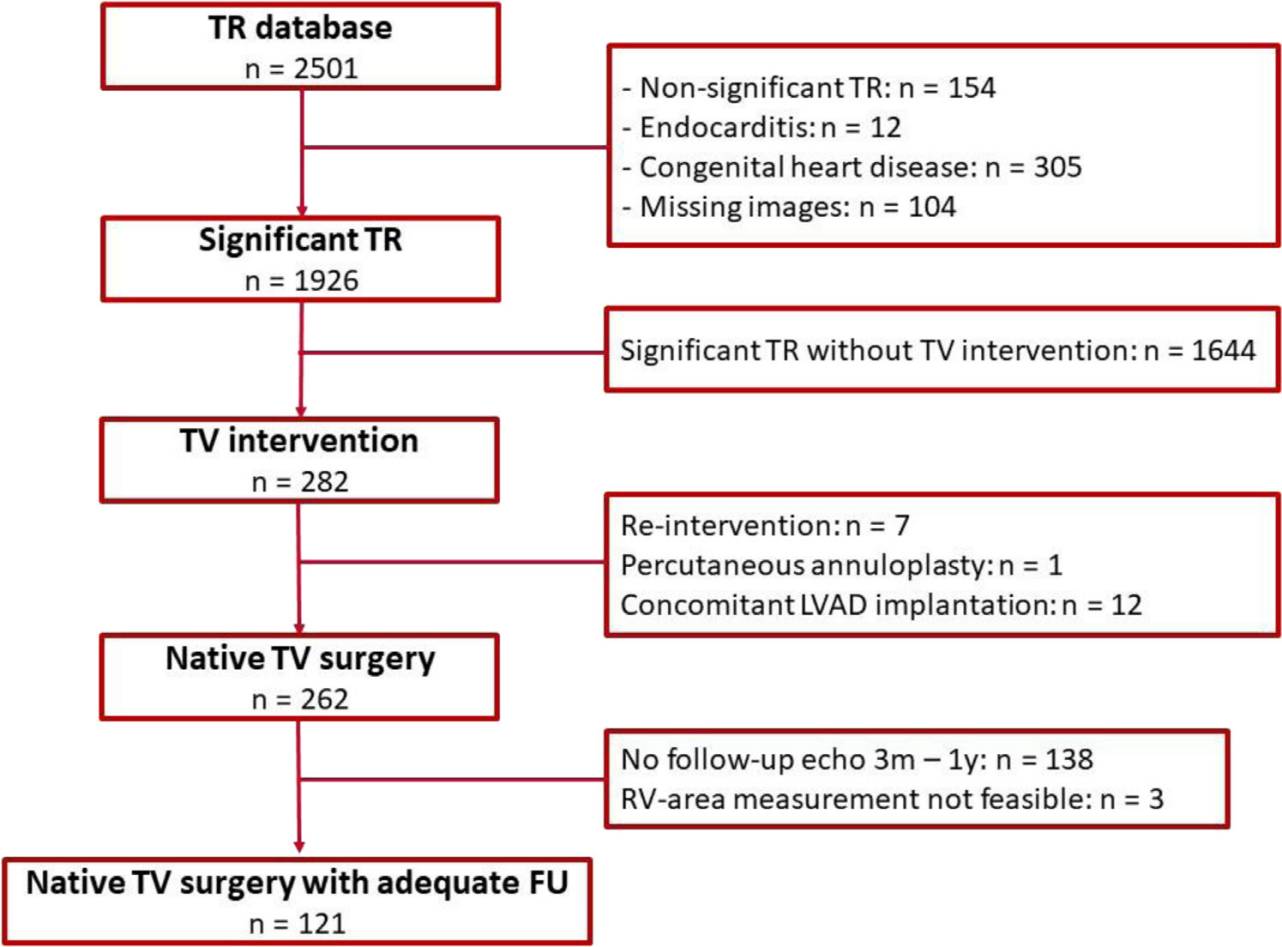
**Flow chart for study population selection.** Abbreviations: FU, follow-up; LVAD, left ventricle assist device; RV, right ventricle; TR, tricuspid regurgitation; TV, tricuspid valve.

Patients with significant TR, diagnosed between January 2000 and September 2016, who subsequently underwent TV surgery, were identified from the departmental echocardiographic database of the Leiden University Medical Center (Leiden, The Netherlands). Significant TR was defined as moderate to severe TR, measured by an integrative approach using qualitative, semiquantitative, and quantitative echocardiographic parameters, as recommended by current guidelines.^19^ Patients aged <18 years or with active endocarditis or known congenital heart disease were excluded from the analysis. In addition, patients with prior TV surgery, percutaneous TV interventions, or concomitant left ventricular assist device implantation, as well as patients with missing or incomplete echocardiographic data were also excluded (Figure 1). Transthoracic echocardiograms were analyzed, and demographic and clinical data were retrospectively collected from the departmental Cardiology Information System (EPD-Vision, Leiden University Medical Center, Leiden, The Netherlands). The institutional review board of the Leiden University Medical Center approved the observational design and retrospective analysis of clinically acquired data and waived the need for patient written informed consent.

### Clinical and Echocardiographic Variables

Baseline demographic, clinical, and laboratory variables were evaluated at the time of TV surgery. Demographic characteristics included age, sex, and body surface area. Clinical characteristics included cardiovascular risk factors, relevant medical history and comorbidities, symptoms of heart failure (New York Heart Association [NYHA] functional class), and heart failure medication.

The baseline echocardiograms, performed prior to surgery, and follow-up echocardiograms, performed 3 months to 1 year after TV surgery, were selected for the analysis. If multiple echocardiograms were available during the prescribed follow-up period, the one closest to 6 months follow-up was used. Transthoracic echocardiograms were performed at rest using commercially available equipment (Vivid 7, E9 and E95 systems, GE-Vingmed, Horten, Norway) and images were digitally stored for offline analysis (EchoPAC version 113.0.3, 202 and 203; GE-Vingmed, Horten, Norway). M-mode, 2-dimensional and color, continuous- and pulsed-wave Doppler data were acquired from the parasternal, apical, and subcostal views according to current guidelines.^19^, ^20^, ^21^, ^22^ From the apical 2- and 4-chamber views, left ventricular ejection fraction was quantified using the biplane Simpson’s method and expressed as a percentage. Left atrial volume was measured at end-systole on the apical 4- and 2-chamber views and indexed for body surface area.^20^ According to current recommendations, mitral valve regurgitation was graded based on qualitative, semiquantitative, and quantitative parameters, evaluated on color, continuous, and pulsed wave Doppler data.^19^^,^^22^ Right atrial dimensions, RV dimensions, and the tricuspid annular end-diastolic diameter were measured on a focused RV apical view. Furthermore, RV systolic function was evaluated by RV fractional area change (RVFAC), derived from the RV end-diastolic and end-systolic areas (RVEDA, RVESA), as well as the tricuspid annular plane systolic excursion (TAPSE) measured on M-mode recordings of the lateral tricuspid annulus on a focused RV apical view. Integrative assessment of TR grade was performed through a multiparametric approach including qualitative, semiquantitative, and quantitative parameters measured on bidimensional, color, and continuous-wave Doppler data of the regurgitant jet, TV morphology, and assessment of the right atrial and RV dimensions, as recommended by current guidelines.^19^ Systolic pulmonary artery pressure was estimated from the TR jet peak velocity, applying the Bernoulli equation and adding right atrial pressure. Right atrial pressure was estimated based on the inferior vena cava diameter and its collapsibility during breathing (≤21 mm with >50% inspiratory collapse: 3 mmHg; >21 mm with ≤50% inspiratory collapse: 15 mmHg; intermediate situations: 8 mmHg).^21^

### Follow-Up and Outcome Definition

The primary study endpoint was all-cause mortality. Follow-up began from the date of follow-up echocardiography after TV surgery. Survival data were ascertained from the departmental Cardiology Information System and the Social Security Death Index.

### Statistical Analysis

Continuous variables with a Gaussian distribution are presented as mean ± standard deviation and continuous variables without a Gaussian distribution are presented as median and interquartile range (IQR). Categorical variables are presented as frequencies and percentages.

Differences between baseline and follow-up echocardiography were analyzed using the paired t-test for continuous variables with normal distribution, and the Wilcoxon signed rank test for nonnormally distributed continuous variables, and the McNemar test for categorical data. To investigate interobserver and variability, in 20 randomly selected patients, the intraclass correlation coefficients were calculated.

**Figure 2:**
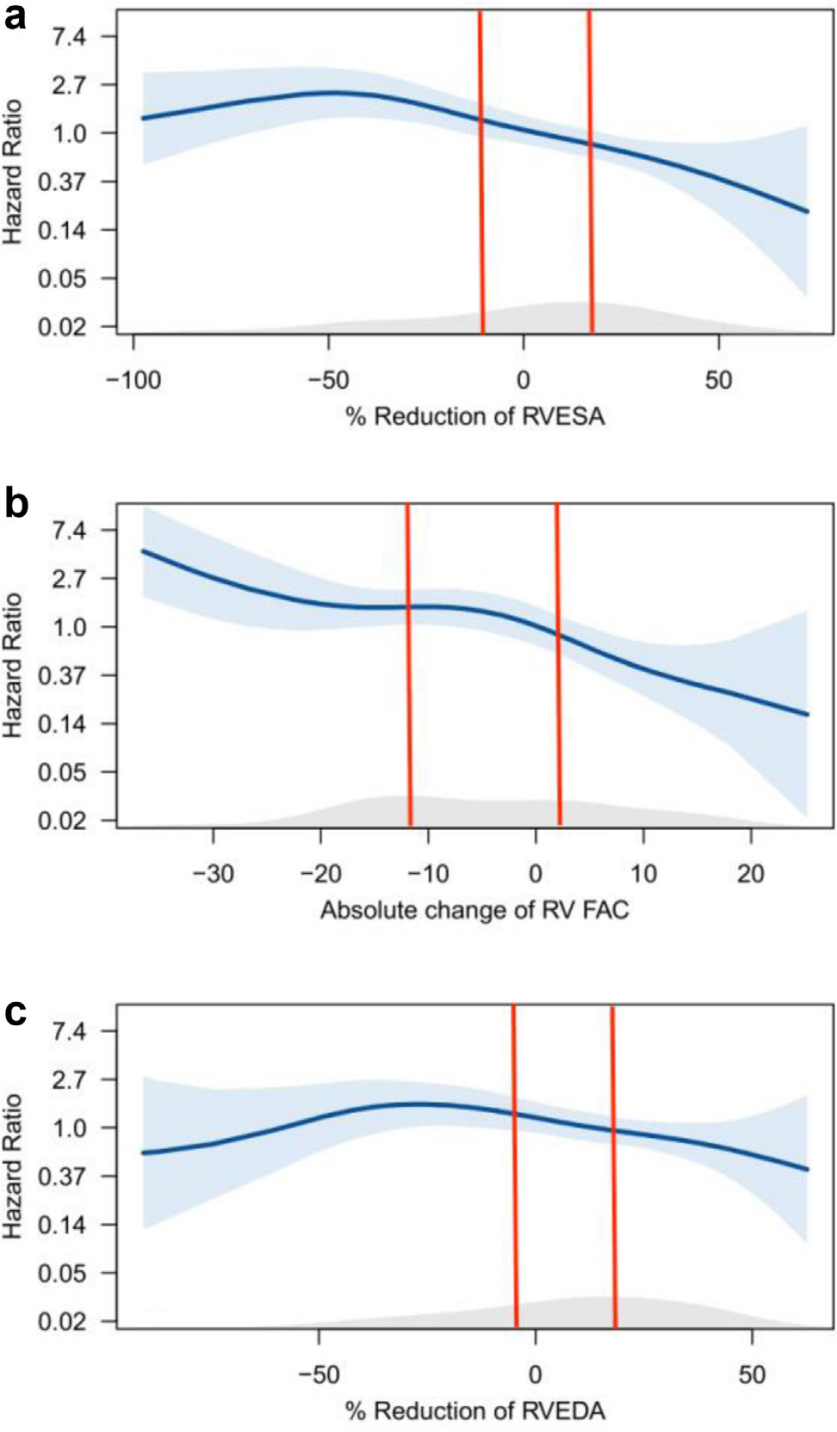
Spline curve plotting the hazard ratio for all-cause mortality according to (a) percentage reduction of right ventricle end-systolic area (RVESA), (b) absolute change of RV fractional area change (RVFAC), and (c) percentage reduction of RV end-diastolic area (RVEDA). The bold blue line represents the spline curve, with overlaid 95% confidence intervals displayed (shaded blue areas). The grey shaded area at the bottom of the panels illustrates the distribution of the population according to (a) percentage reduction of RVESA, (b) absolute change of RVFAC, and (c) percentage reduction of RVEDA. Vertical red lines mark the tertiles of the overall population. Abbreviation: RV, right ventricle.

RV reverse remodeling was assessed according to changes in RV dimensions (RVESA and RVEDA), as well as RV function (RVFAC). To investigate the association between RV reverse remodeling and all-cause mortality, a spline curve was fitted in an unadjusted model (Figure 2), demonstrating the hazard ratio for all-cause mortality according to the percentage reduction of RVESA (Figure 2a), absolute change of RVFAC (Figure 2b), and percentage reduction of RVEDA (Figure 2c), respectively. Based on these spline curve analyses, the population was stratified by tertiles of percentage reduction of RV area and absolute change of RVFAC to provide insight into patients with an improvement, stabilization, or worsening of the RV size and/or RV function. The Kaplan–Meier survival analysis was used to estimate the 5-year survival rate, and differences between groups were analyzed using a log-rank test. To identify patients unlikely to present RV reverse remodeling, the association between baseline/procedural factors and the absence of echocardiographic RV reverse remodeling (corresponding to tertile 1 for both RVESA and RVFAC) was investigated by performing univariable and multivariable Cox proportional hazards regression analyses. Moreover, univariable and multivariable Cox proportional hazards regression analyses were performed to assess the clinical and echocardiographic factors that were independently associated with all-cause mortality. Variables that were significant in the univariable analysis were selected for multivariable regression analysis. To avoid overfitting the multivariable model, and since the aim of the study was to investigate the prognostic implications of RV reverse remodeling (assessed according to change in RV area or RVFAC) in patients undergoing TV surgery for significant TR, different models have been constructed to evaluate the association of RV reverse remodeling, each time corrected for 2 out of 3 variables that were significant on univariable analysis. Results are provided for RV area and RVFAC, treated as a continuous variable as well as a categorical variable stratified into tertiles. In addition, variables included in the multivariable regression analysis had less than 5% missing values. Correlation factor analysis was used to determine if any pairs of variables were correlated and no collinearity (correlation coefficient of >0.60) was detected for the variables that met the entry criteria for multivariable regression analysis. Hazard ratios and 95% confidence intervals were calculated. All *p*-values were 2-sided, and values < 0.05 were considered significant. All data were analyzed using SPSS for Windows, version 23 (SPSS Inc, IBM Corp, Armonk, New York) and R version 4.0.1 (R Foundation for Statistical Computing, Vienna, Austria).

## Results

### Clinical Characteristics

A total of 121 patients (63 ± 12 years, 47% males) were included in the analysis. Clinical characteristics of the overall population are presented in Table 1. A large proportion of patients had arterial hypertension (64%), atrial fibrillation (56%), and symptoms of heart failure (61% had NYHA functional class III or IV). A total of 45 (37%) patients had a pacemaker or implantable cardioverter-defibrillator and 28 (23%) patients had previous cardiac surgery. Most patients (97%) underwent TV annuloplasty, the majority being concomitant to left-sided valve surgery (75% mitral valve surgery and 27% aortic valve surgery) or coronary artery bypass grafting (16%).

**Table 1:** Baseline characteristics of the total population

Variable	Overall population (n = 121)
Demographic characteristics	
Age, y	63 ± 12
Male gender	57 (47)
Body mass index, kg/m^2^	25.2 ± 3.7
Medical history	
Arterial hypertension	77 (64)
Dyslipidemia	46 (38)
Diabetes mellitus	18 (15)
Smoking	49 (41)
Coronary artery disease	39 (32)
Atrial fibrillation	68 (56)
Pacemaker/ICD	45 (37)
Previous cardiac surgery	28 (23)
Chronic kidney disease	29 (29)
COPD	13 (11)
NYHA III or IV	72 (61)
Laboratory values	
Hemoglobin, mmol/L	8.1 ± 1.3
Creatinine, μmol/L	93 (77-124)
Medication	
Beta-blocker	75 (62)
ACE-inh/ARB	85 (70)
Loop diuretic	94 (78)
MRA	49 (41)
Statin	53 (44)
Surgical characteristics	
Tricuspid valve annuloplasty	117 (97)
Concomitant surgery	109 (90)
Concomitant CABG	19 (16)
Concomitant MV surgery	91 (75)
Concomitant AV surgery	33 (27)

*Notes*. Values are mean ± SD, median (IQR), or n (%).

ACE-inh, angiotensin-converting enzyme inhibitor; ARB, angiotensin receptor blocker; AV, aortic valve; CABG, coronary artery bypass grafting; COPD, chronic obstructive pulmonary disease; ICD, implantable cardioverter-defibrillator; IQR, interquartile range; MRA, mineralocorticoid receptor antagonist; MV, mitral valve; NYHA, New York Heart Association functional class.

Spline curve analysis (Figure 2) showed that a reduction of RVESA and improvement in RVFAC were significantly associated with better survival after TV surgery, whereas the association between percentage reduction of RVEDA and the primary outcome was not statistically significant. Therefore, no further analyses were performed based on the change of RVEDA and the population was stratified by tertiles of percentage reduction of RVESA and absolute change of RVFAC only. Cutoff values identified to define RV remodeling tertiles were +11.2% relative increase and −17.2% relative decrease of RVESA together with −11.7% absolute decrease and +2.3% absolute increase of RVFAC. The clinical characteristics of the population were stratified according to RVESA- and RVFAC-tertiles in the Supplemental Tables 1 and 2, respectively.

### Echocardiographic Characteristics

Table 2 summarizes the baseline and follow-up echocardiographic characteristics. Follow-up echocardiogram after TV intervention was performed after a mean follow-up of 7 ± 3 months. Dividing the study population according to early or late follow-up (cutoff was based on the median time between TV surgery and follow-up echocardiogram; as well as according to tertiles), no significant differences in RV reverse remodeling was found between the groups (Supplemental Tables 3 and 4).

**Table 2:** Echocardiographic characteristics of the total population at baseline and at follow-up

Variable	Baseline echocardiogram (n = 121)	Follow-up echocardiogram (n = 121)	*p*-value
Heart rate			
Heart rate, beats/min	82 ± 16	76 ± 14	0.001
LV, LA, and left-sided valvular disease			
LV end-diastolic volume, mL	148 (87-203)	134 (96-180)	0.078
LV end-systolic volume, mL	83 (43-138)	73 (47-121)	0.387
LV ejection fraction, %	42 ± 15	41 ± 15	0.646
LA end-systolic volume – indexed, mL/m^2^	55 (38-80)	48 (34-69)	<0.001
Moderate and severe mitral regurgitation	88 (73)	19 (16)	<0.001
RV and RA			
RV basal diameter, mm	49 ± 10	45 ± 9	<0.001
RV mid diameter, mm	38 ± 10	34 ± 8	<0.001
RV length, mm	79 ± 14	79 ± 14	0.732
RV end-diastolic area, cm^2^	27 ± 10	24 ± 8	<0.001
RV end-systolic area, cm^2^	18 ± 8	17 ± 8	0.312
RV fractional area change, %	35 ± 11	31 ± 12	<0.001
TAPSE, mm	17 ± 5	12 ± 4	<0.001
RV peak systolic pressure, mm Hg	46 (34-59)	30 (11-44)	<0.001
Right atrial maximum area, cm^2^	27 (21-35)	21 (16-25)	<0.001
Tricuspid valve disease			
Moderate and severe tricuspid regurgitation	121 (100)	32 (26)	<0.001
Tricuspid valve annular diameter, mm	43 ± 8	34 ± 12	<0.001

*Notes*. Values are mean ± SD, median (IQR), or n (%).

LA, left atrium; LV, left ventricle; RA, right atrium; RV, right ventricle; TAPSE, tricuspid annular plane systolic excursion.

The baseline mean left ventricular ejection fraction was 42 ± 15%, and concomitant significant mitral regurgitation was present in 88 (73%) patients. In the overall population, the RV was dilated at baseline. Moreover, baseline RV systolic function was on average normal, with a mean RVFAC of 35 ± 11% and a mean TAPSE of 17 ± 5 mm. RV systolic pressure was elevated with a median of 46 (IQR: 34-59) mmHg.

At follow-up, there was no significant difference in left ventricular dimensions or left ventricular ejection fraction. A significant reduction in mitral regurgitation and consequently reduction in left atrial volume was observed. There was a significant reduction in most of the right heart variables after TV surgery. Particularly significant decreases in TV annular, RV basal, and RV mid-diameters as well as RVEDA together with a significant decrease in RV systolic function were observed. Furthermore, there was a significant decrease in RV peak systolic pressure along with a significant decrease in right atrial maximum area.

Interobserver reproducibility showed good agreement for RVEDA and RVESA, with moderate and good agreement for RVFAC and TAPSE, respectively. The intraobserver reproducibility showed good agreement for RVFAC and excellent agreement for other variables (Supplemental Tables 5 and 6).

### Prognostic Impact of RV Reverse Remodeling

**Figure 3:**
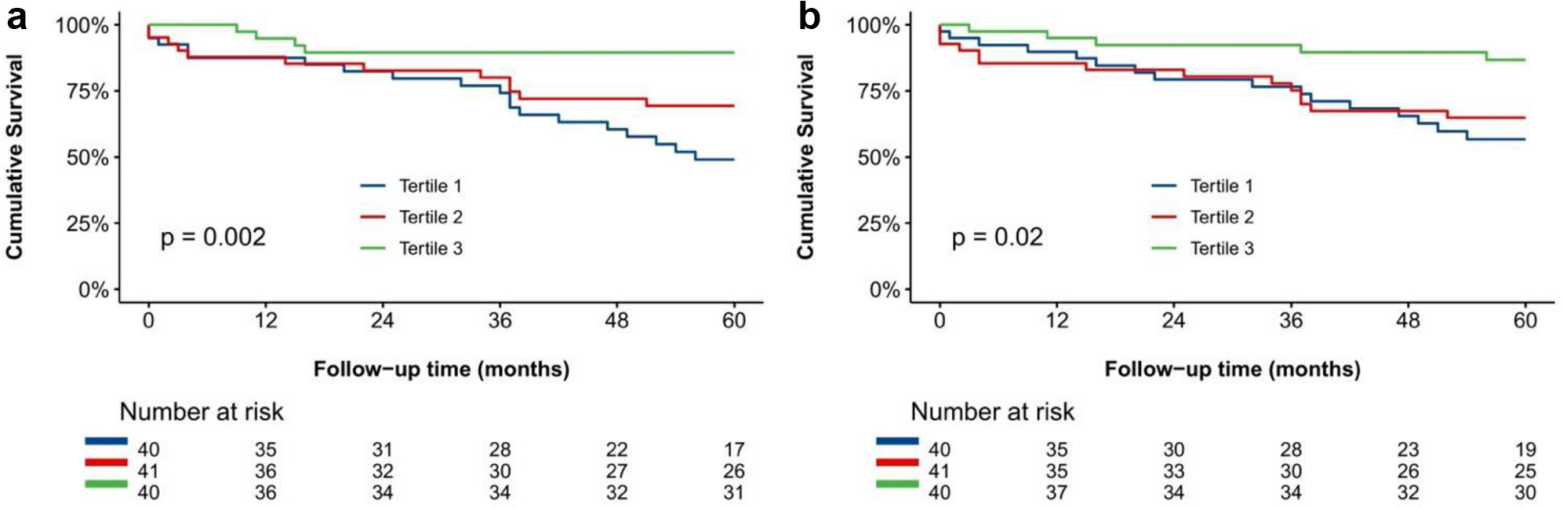
Kaplan-Meier curves for overall survival according to (a) right ventricle (RV) end-systolic area-tertiles and (b) RV fractional area change-tertiles. The blue, red, and green curves demonstrate the Kaplan-Meier curves for overall survival, respectively, for tertile 1, tertile 2, and tertile 3.

Over a median follow-up of 75 (IQR: 37-109) months, 53 (44%) patients died. The 1- and 5-year cumulative survival rates were 90% and 70%, respectively, for the total population; with 35 deaths at 5-year follow-up. Figure 3 shows the Kaplan-Meier analysis for all-cause mortality according to the RVESA-tertiles (Figure 3a) and RVFAC-tertiles (Figure 3b). Survival rates at 5-year follow-up were significantly better in patients with more pronounced RV reverse remodeling, assessed according to RVESA change: 49%, 69%, and 90% for tertile 1, tertile 2, and tertile 3 (log-rank chi-square: 12.526; *p* = 0.002), respectively; as well as according to RVFAC change: 57%, 65%, and 87% for tertile 1, tertile 2, and tertile 3 (log-rank chi-square: 7.784; *p* = 0.02), respectively.

Univariable and multivariable Cox proportional hazards regression analyses for the absence of echocardiographic RV reverse remodeling are presented in the supplementary material (Supplemental Tables 7 and 8, respectively). For RVESA, on univariable analysis the presence of a NYHA class III or IV was associated with the absence of remodeling, whereas larger RVEDA and larger RVESA were associated with more remodeling. NYHA class III or IV and RVEDA remained independently associated with RV reverse remodeling on multivariable analysis, even after correcting for age and gender. For RVFAC, none of the variables were significantly associated with the absence of RV reverse remodeling on univariable analysis.

To investigate the association between echocardiographic RV reverse remodeling and all-cause mortality, univariable and multivariable Cox proportional hazards regression analyses were performed (Tables 3 and 4, respectively). On univariable analysis, diabetes mellitus and pacemaker or implantable cardioverter-defibrillator were associated with significantly worse overall survival. Conversely, baseline TAPSE and RV reverse remodeling assessed by RVESA change as well as RVFAC change were associated with significantly better overall survival. Patients within tertile 3 had significantly better survival compared to those in tertile 1 for RVESA change (hazard ratio: 0.175 [95% CI: 0.060-0.516]) and for RVFAC change (hazard ratio: 0.265 [95% CI: 0.097-0.723]). On multivariable analysis, following adjustment for the significant variables on univariable analysis, tertile 3 of RVESA and RVFAC changes remained independently associated with better survival (Table 4).

**Table 3:** Univariable Cox proportional hazard models for all-cause mortality in patients with significant tricuspid regurgitation who underwent tricuspid valve surgery

Variable	Univariable analysis	
	Hazard ratio (95% CI)	*p*-value
Age, y	1.002 (0.974-1.030)	0.915
Male gender	1.083 (0.558-2.102)	0.813
Arterial hypertension	0.588 (0.302-1.145)	0.119
Dyslipidemia	1.563 (0.805-3.035)	0.187
Diabetes mellitus	2.242 (1.049-4.789)	0.037
Smoking	1.058 (0.541-2.066)	0.870
Coronary artery disease	1.360 (0.692-2.675)	0.373
Atrial fibrillation	0.814 (0.419-1.579)	0.542
Pacemaker/ICD	3.667 (1.844-7.293)	<0.001
Previous cardiac surgery	0.990 (0.450-2.179)	0.980
COPD	1.497 (0.581-3.861)	0.403
NYHA III or IV	1.448 (0.709-2.957)	0.309
Loop diuretic	2.475 (0.873-7.017)	0.088
Hemoglobin, mmol/L	1.056 (0.803-1.388)	0.699
Creatinine, μmol/L	1.002 (0.998-1.006)	0.257
Tricuspid valve annuloplasty	1.087 (0.148-7.959)	0.935
Concomitant surgery	0.746 (0.263-2.116)	0.581
Left ventricular ejection fraction, %	0.979 (0.957-1.001)	0.062
LA end-systolic volume – indexed, mL/m^2^	1.000 (0.993-1.006)	0.885
RV basal diameter, mm	1.018 (0.982-1.055)	0.335
RV mid diameter, mm	1.017 (0.984-1.052)	0.312
RV length, mm	1.002 (0.978-1.027)	0.847
RV end-diastolic area, mm^2^	0.999 (0.966-1.033)	0.940
RV end-systolic area, mm^2^	0.996 (0.951-1.043)	0.866
RV fractional area change, %	1.004 (0.972-1.037)	0.798
TAPSE, mm	0.896 (0.826-0.971)	0.007
RV peak systolic pressure, mmHg	0.983 (0.963-1.003)	0.093
Right atrial maximum area, mm^2^	0.999 (0.967-1.032)	0.941
Tricuspid valve annular diameter, mm	1.011 (0.970-1.053)	0.614
RV end-systolic area reduction (Δ) - continuous	0.992 (0.987-0.997)	0.003
RV end-systolic area reduction (Δ) - tertiles		0.005
Tertile 1: Δ < −11.2%	Ref.	Ref.
Tertile 2: −11.2% < Δ < 17.2%	0.579 (0.281-1.194)	0.139
Tertile 3: Δ > 17.2%	0.175 (0.060-0.516)	0.002
RV fractional area change (Δ) – continuous	0.959 (0.935-0.983)	0.001
RV fractional area change (Δ) – tertiles		0.032
Tertile 1: Δ < −11.7%	Ref.	Ref.
Tertile 2: −11.7% < Δ < 2.3%	0.829 (0.404-1.699)	0.609
Tertile 3: Δ > 2.3%	0.265 (0.097-0.723)	0.010

Δ, change/reduction in RV end-systolic area or RV fractional area change; CI, confidence interval; COPD, chronic obstructive pulmonary disease; ICD, implantable cardioverter-defibrillator; LA, left atrium; NYHA, New York Heart Association functional class; RV, right ventricle; TAPSE, tricuspid annular plane systolic excursion.

**Table 4:** Multivariable Cox proportional hazard models for all-cause mortality for RV end-systolic area and RV fractional area change, following adjustment in each model for 2 out of 3 variables that were significant on univariable analysis (diabetes mellitus, CIED and TAPSE)

Variable	Multivariable analysis Model 1		Multivariable analysis Model 2		Multivariable analysis Model 3	
	Hazard ratio (95% CI)	*p*-value	Hazard ratio (95% CI)	*p*-value	Hazard ratio (95% CI)	*p*-value
RV end-systolic area reduction - continuous	0.993 (0.988-0.999)	0.015	0.993 (0.988-0.998)	0.011	0.993 (0.988-0.999)	0.016
RV end-systolic area reduction - tertiles		0.028		0.013		0.021
Tertile 1	Ref.	Ref.	Ref.	Ref.	Ref.	Ref.
Tertile 2	0.661 (0.318-1.375)	0.268	0.620 (0.300-1.280)	0.196	0.666 (0.320-1.385)	0.277
Tertile 3	0.229 (0.077-0.685)	0.008	0.201 (0.068-0.596)	0.004	0.218 (0.073-0.645)	0.006
RV fractional area change – continuous	0.965 (0.942-0.988)	0.003	0.964 (0.939-0.989)	0.005	0.964 (0.940-0.988)	0.004
RV fractional area change - tertiles		0.051		0.103		0.026
Tertile 1	Ref.	Ref.	Ref.	Ref.	Ref.	Ref.
Tertile 2	0.925 (0.441-1.943)	0.837	0.895 (0.436-1.839)	0.763	0.993 (0.467-2.113)	0.986
Tertile 3	0.294 (0.107-0.811)	0.018	0.338 (0.122-0.936)	0.037	0.322 (0.117-0.889)	0.029

*Notes*. Model 1: + diabetes mellitus + TAPSE; Model 2: + diabetes mellitus + CIED; Model 3: + CIED + TAPSE.

CI, confidence interval; CIED, cardiac implantable electronic device; RV, right ventricle; TAPSE, tricuspid annular plane systolic excursion.

## Discussion

The main findings of the present study are 2-fold: (i) patients treated with TV surgery for significant TR present with significant right atrial and RV reverse remodeling, and (ii) the magnitude of RV reverse remodeling based on the percentage reduction of RVESA together with the improvement of absolute change in RVFAC was significantly associated with 5-year overall survival, with tertile 3 for RVESA relative reduction as well as for RVFAC absolute improvement being independently associated with better outcomes after TV surgery compared to tertile 1.

### RV Reverse Remodeling After TV Surgery

Current guidelines recommend surgical TV repair in patients with symptomatic severe TR and in patients with a dilated TV annulus who undergo left-sided valve surgical intervention.^7^^,^^8^ The beneficial effect of TV surgery on TR severity as well as TR recurrence has been demonstrated in several studies.^11^^,^^14^^,^^23^^,^^24^ However, inconsistent changes in RV dimensions and function after TV surgery have been reported.

In a cohort of 45 patients undergoing mitral valve repair and concomitant TV surgery for severe TR and/or a lower TR grade with a dilated TV annulus, Bertrand et al.^12^ described no changes in RVEDA and a nonsignificant decrease in RVFAC but a significant increase in the RV sphericity index in the overall population. However, a subanalysis based on baseline TR severity showed that patients undergoing TV surgery for moderate or less TR but with tricuspid annular dilatation had unchanged RVEDA and RVFAC after surgery, whereas patients undergoing TV surgery for severe TR presented a significant decrease in RVEDA and RVFAC. These results led the authors to conclude that the extent of RV reverse remodeling after TV surgery is directly proportional to the extent of RV volume overload before surgery. These findings are in line with the findings described by Van de Veire et al. and Kim et al.^11^^,^^17^ The results of the present study, including patients undergoing TV surgery for significant TR, further confirms this hypothesis.

Discrepant changes in RV systolic function have been observed after TV surgery. The complex 3-dimensional RV geometry, which has a triangular shape in the coronal plane and a crescent shape in the transverse plane, makes it challenging to accurately evaluate RV function. Moreover, evaluating RV systolic function by RVFAC relies on geometric assumptions and is load dependent. Therefore, in the presence of significant TR, RV contractile function assessed by RVFAC may be overestimated due to the increased preload.^25^ Treating significant TR with TV surgery will reduce RV preload and consequently reduce RV dimensions. However, RV end-diastolic dimensions are more preload-dependent while RV end-systolic dimensions are more afterload-dependent. As a result, RVEDA will decrease to a greater extent than RVESA after TV surgery for significant TR. Similarly, in the present study, including patients with significant TR, a significant decrease in RVEDA with unchanged RVESA was observed, resulting in a significant decrease in RVFAC. These findings are in agreement with the findings reported by Bertrand et al. and Kim et al., both with similar echocardiographic follow-up of 5 and 6 months, respectively.^12^^,^^18^ Studies evaluating echocardiographic changes in RV systolic function at longer follow-up (ranging from 1 to 5 years) have described unchanged or even improved RVFAC, indicating ongoing RV reverse remodeling after TV surgery.^15^, ^16^, ^17^

### Prognostic Impact of RV Reverse Remodeling

Untreated, severe TR is associated with poor survival, and RV dilatation together with RV dysfunction are independent predictors of prognosis in patients with medically treated severe TR.^1^^,^^3^^,^^4^ Nevertheless, still few patients are referred for TV surgery since timing of surgical intervention remains challenging and surgical indication is still largely driven by the primary indication for left-sided valve surgery. Moreover, various registries have reported poor operative outcomes of isolated TV surgery, ranging around 10%, which is significantly higher when compared to those of other valve interventions.^18^^,^^26^^,^^27^ Preoperative RV dimensions (TV annular diameter and RVESA) as well as RV systolic dysfunction are independent correlates of survival after isolated and concomitant TV surgical intervention.^3^^,^^5^^,^^13^^,^^18^ Moreover, Park et al.^6^ have shown that postoperative RV systolic function in patients undergoing surgery for isolated severe TR is also a predictor of long-term event-free survival. However, the association of postoperative RV reverse remodeling, based on changes in RV dimensions and function, with survival has not been extensively explored. In a cohort of 90 patients who had isolated TV surgery, Patlolla et al.^28^ showed that RV reverse remodeling (defined as normalization in RV size and function) was independently associated with improved survival (hazard ratio: 0.42 [95%CI: 0.24-0.74]). To the best of our knowledge, the present study is the first reporting on the association of RV reverse modeling and outcomes after TV surgery in a population with the majority of patients undergoing TV surgery concomitant to left-sided heart surgery. The extent of the decrease in RVESA or improvement in RVFAC was independently associated with the outcome after TV intervention.

### Clinical Implications

Echocardiographic follow-up after heart valve surgery is important to ensure durability of the valve repair and restoration of the cardiac hemodynamics after relief of pressure and volume overload.^7^^,^^8^ There is a need for an intensive and comprehensive follow-up of these patients after cardiac surgery. This study evaluated the prognostic value of RV reverse remodeling, according to RVESA and RVFAC, after TV surgery. Patients presenting with significant RV reverse remodeling, based on a relative reduction in RVESA or absolute improvement in RVFAC, have significant better outcome compared to patients who do not show evidence of such a RV reverse remodeling. Accordingly, the latter patients may benefit from a continued intensive follow-up for further adjustment of heart failure medical therapy.

### Study Limitations

Several limitations of the present study need to be acknowledged. First, this study is limited by its retrospective design from a single tertiary center. The retrospective nature contributes to the variability in timing of follow-up echocardiography, which may influence the results of remodeling of the right ventricle. Subanalyses for early vs. late inclusion showed, however, no significant differences in remodeling according to the timing of follow-up echocardiography. Second, the number of patients was limited and the results need to be confirmed in larger, prospective cohorts. Third, all patients included in the present study completed follow-up echocardiogram. Hence, patients who died before this follow-up could not be included and may have caused a selection bias and immortal time bias. Fourth, the complex 3-dimensional geometry of the RV limits an accurate 2-dimensional echocardiographic evaluation of RV size and function. Test-retest reproducibility for the presented data in this study, however, was moderate to good and good to excellent for interobserver and intraobserver reproducibility, respectively. Last, left-sided valvular disease etiologies, as well as the variability in the type of concomitant left-sided surgery, may lead to differences in outcome.

## Conclusion

The majority of patients undergoing TV surgery for significant TR presented significant RV reverse remodeling. The magnitude of RV reverse remodeling, based on a reduction of RVESA and an improvement of absolute change in RVFAC, was associated with significantly better overall survival at 5-year follow-up after TV surgery for significant TR.

## Ethics Statement

The present research has adhered to the relevant ethical guidelines. Informed consent was waived by the Institutional Review Board due to the retrospective nature of the study.

## Funding

The authors have no funding to report.

## Disclosure statement

The Department of Cardiology, Heart Lung Center, Leiden University Medical Centre has received research grants from Abbott Vascular, Bayer, Biotronik, Bioventrix, Boston Scientific, Edwards Lifesciences, GE Healthcare, Ionis, Medtronic, and Novartis. J.S. received funding from the European Society of Cardiology (ESC Training Grant App000064741). N.A.M. received speaker fees from Abbott Vascular and GE Healthcare. J.J.B. received speaker fees from Abbott Vascular and Edwards Lifesciences. V.D. received speaker fees from Abbott Vascular, Edwards Lifesciences, GE Healthcare, Medtronic, MSD, and Novartis. The other authors had no conflicts to declare.
