# Supplementary material for: Right Ventricular Reverse Remodeling After Tricuspid Valve Surgery for Significant Tricuspid Regurgitation
Source: Struct Heart. 2022 Nov 4;7(1):100101. doi: 10.1016/j.shj.2022.100101 (PMC10236803; doi:10.1016/j.shj.2022.100101)
Supplement: Supplemental Tables 1-8 [file mmc1.docx]

**Supplementary material**

**Table S1.** Baseline characteristics of the total population and according to tertiles of right ventricular remodeling defined by the relative reduction in RVESA.

| **RVESA** | **Overall population**  **(n = 121)** | **Tertile 1**  **(n = 40)** | **Tertile 2**  **(n= 41)** | **Tertile 3**  **(n= 40)** | **p-value** |
| --- | --- | --- | --- | --- | --- |
| **Demographic characteristics** | | | | | |
| Age, years | 63 ± 12 | 64 ± 14 | 64 ± 10 | 62 ± 1 | 0.712 |
| Male gender | 57 (47) | 20 (50) | 17 (42) | 20 (50) | 0.673 |
| Body Mass index, kg/m^2^ | 25.2 ± 3.7 | 24.8 ± 3.2 | 24.3 ± 3.2 | 26.5 ± 4.3§ | 0.017 |
| **Medical history** | | | | | |
| Arterial hypertension | 77 (64) | 20 (50) | 28 (68) | 29 (73) | 0.084 |
| Dyslipidemia | 46 (38) | 18 (45) | 11 (27) | 17 (43) | 0.188 |
| Diabetes mellitus | 18 (15) | 8 (20) | 7 (17) | 3 (8) | 0.259 |
| Smoking | 49 (41) | 14 (35) | 15 (37) | 20 (50) | 0.323 |
| Coronary artery disease | 39 (32) | 14 (35) | 11 (27) | 14 (35) | 0.661 |
| Atrial fibrillation | 68 (56) | 23 (58) | 27 (66) | 18 (45) | 0.164 |
| Pacemaker/ICD | 45 (37) | 16 (40) | 17 (42) | 12 (30) | 0.511 |
| Previous cardiac surgery | 28 (23) | 8 (20) | 15 (37) | 5 (13)§ | 0.031 |
| Chronic kidney disease | 29 (29) | 15 (47) | 9 (27) | 5 (15)* | 0.015 |
| COPD | 13 (11) | 5 (13) | 4 (10) | 4 (10) | 0.908 |
| NYHA III or IV | 72 (61) | 29 (73) | 23 (58) | 20 (51) | 0.139 |
| **Laboratory values** | | | | | |
| Hemoglobin, mmol/L | 8.1 ± 1.3 | 8.2 ± 1.4 | 7.7 ± 1.3 | 8.4 ± 1.0 | 0.060 |
| Creatinine, µmol/L | 93 (77 - 124) | 110 (78 - 131) | 94 (79 - 139) | 84 (74 - 103) | 0.104 |
| **Medication** | | | | | |
| Beta-blocker | 75 (62) | 29 (73) | 22 (54) | 24 (60) | 0.207 |
| ACE-inh / ARB | 85 (70) | 25 (63) | 31 (77) | 29 (73) | 0.405 |
| Loop diuretic | 94 (78) | 30 (75) | 33 (81) | 31 (78) | 0.838 |
| MRA | 49 (41) | 16 (40) | 18 (44) | 15 (38) | 0.839 |
| Statin | 53 (44) | 21 (53) | 16 (39) | 16 (40) | 0.398 |
| **Surgical characteristics** | | | | | |
| Tricuspid valve annuloplasty | 117 (97) | 40 (100) | 39 (95) | 38 (95) | 0.360 |
| Concomitant surgery | 109 (90) | 37 (93) | 37 (90) | 35 (88) | 0.755 |
| Concomitant CABG | 19 (16) | 8 (20) | 7 (17) | 4 (10) | 0.450 |
| Concomitant MV surgery | 91 (75) | 30 (75) | 34 (83) | 27 (68) | 0.275 |
| Concomitant AV surgery | 33 (27) | 8 (20) | 17 (42) | 8 (20) | 0.043 |

Values are mean ± SD, median (IQR), or n (%). *p<0.05 vs tertile 1 and §p<0.05 vs tertile 2.

ACE-inh, angiotensin-converting enzyme inhibitor; ARB, angiotensin receptor blocker; AV, aortic valve; CABG, coronary artery bypass grafting; COPD, chronic obstructive pulmonary disease; ICD, implantable cardioverter-defibrillator; IQR, interquartile range; MRA, mineralocorticoid receptor antagonist; MV, mitral valve; NYHA, New York Heart Association functional class; and RVESA, right ventricular end-systolic area.

**Table S2.** Baseline characteristics of the total population and according to tertiles of right ventricular remodeling defined by absolute change in RVFAC.

| **RVFAC** | **Overall population**  **(n = 121)** | **Tertile 1**  **(n = 40)** | **Tertile 2**  **(n= 41)** | **Tertile 3**  **(n= 40)** | **p-value** |
| --- | --- | --- | --- | --- | --- |
| **Demographic characteristics** | | | | | |
| Age, years | 63 ± 12 | 65 ± 11 | 64 ± 13 | 62 ± 10 | 0.454 |
| Male gender | 57 (47) | 19 (48) | 19 (46) | 19 (48) | 0.993 |
| Body Mass index, kg/m^2^ | 25.2 ± 3.7 | 25.4 ± 3.6 | 24.9 ± 3.4 | 25.4 ± 4.1 | 0.794 |
| **Medical history** | | | | | |
| Arterial hypertension | 77 (64) | 29 (73) | 21 (51) | 27 (68) | 0.114 |
| Dyslipidemia | 46 (38) | 11 (28) | 19 (46) | 16 (40) | 0.207 |
| Diabetes mellitus | 18 (15) | 8 (20) | 6 (15) | 4 (10) | 0.453 |
| Smoking | 49 (41) | 16 (40) | 17 (42) | 16 (40) | 0.988 |
| Coronary artery disease | 39 (32) | 13 (33) | 16 (39) | 10 (25) | 0.401 |
| Atrial fibrillation | 68 (56) | 24 (60) | 20 (49) | 24 (60) | 0.500 |
| Pacemaker/ICD | 45 (37) | 18 (45) | 16 (39) | 11 (28) | 0.258 |
| Previous cardiac surgery | 28 (23) | 10 (25) | 9 (22) | 9 (23) | 0.942 |
| Chronic kidney disease | 29 (29) | 12 (34) | 9 (27) | 8 (25) | 0.680 |
| COPD | 13 (11) | 6 (15) | 3 (7) | 4 (10) | 0.527 |
| NYHA III or IV | 72 (61) | 25 (64) | 26 (63) | 21 (54) | 0.583 |
| **Laboratory values** | | | | | |
| Hemoglobin, mmol/L | 8.1 ± 1.3 | 8.2 ± 1.2 | 8.1 ± 1.5 | 8.0 ± 1.2 | 0.848 |
| Creatinine, µmol/L | 93 (77 - 124) | 93 (79 - 122) | 97 (77 - 125) | 91 (69 - 129) | 0.867 |
| **Medication** | | | | | |
| Beta-blocker | 75 (62) | 30 (75) | 25 (61) | 20 (50) | 0.070 |
| ACE-inh / ARB | 85 (70) | 32 (80) | 29 (71) | 24 (60) | 0.147 |
| Loop diuretic | 94 (78) | 35 (88) | 30 (73) | 29 (73) | 0.190 |
| MRA | 49 (41) | 16 (40) | 18 (44) | 15 (38) | 0.839 |
| Statin | 53 (44) | 16 (40) | 24 (59) | 13 (33) | 0.052 |
| **Surgical characteristics** | | | | | |
| Tricuspid valve annuloplasty | 117 (97) | 38 (95) | 39 (95) | 40 (100) | 0.360 |
| Concomitant surgery | 109 (90) | 34 (85) | 36 (88) | 39 (98) | 0.145 |
| Concomitant CABG | 19 (16) | 7 (18) | 5 (12) | 7 (18) | 0.750 |
| Concomitant MV surgery | 91 (75) | 27 (68) | 30 (73) | 34 (85) | 0.181 |
| Concomitant AV surgery | 33 (27) | 11 (28) | 11 (27) | 11 (28) | 0.997 |

Values are mean ± SD, median (IQR), or n (%). *p<0.05 vs tertile 1 and §p<0.05 vs tertile 2.

ACE-inh, angiotensin-converting enzyme – inhibitor; ARB, angiotensin receptor blocker; AV, aortic valve; CABG, coronary artery bypass grafting; COPD, chronic obstructive pulmonary disease; ICD, implantable cardioverter-defibrillator; IQR, interquartile range; MRA, mineralocorticoid receptor antagonist; MV, mitral valve; NYHA, New York Heart Association functional class; and RVFAC, right ventricular fractional area change.

**Table S3.** Baseline values for RV end-systolic area and RV fractional area change as well as remodeling (percentage of reduction in RV end-systolic area and absolute change RV fractional area change) according to an early or late inclusion (split by the median time of follow-up echocardiography after surgery).

| **Total population** | **Early inclusion < median FU time**  **n = 62** | | | **Late inclusion > median FU time**  **n = 59** | | | **RV reverse remodeling** | | | |
| --- | --- | --- | --- | --- | --- | --- | --- | --- | --- | --- |
|  | **Baseline echocardiogram** | **Follow-up echocardiogram** | **p-value** | **Baseline echocardiogram** | **Follow-up echocardiogram** | **p-value** | **Early inclusion** | **Late inclusion** | **p-value** |  |
| RV end-systolic area, cm^2^ | 18 ± 7 | 18 ± 7 | 0.264 | 17 ± 8 | 17 ± 8 | 0.659 | -1.37 ± 33.76 | -8.71 ± 57.48 | 0.397 |  |
| RV fractional area change, % | 34 ± 10 | 30 ± 11 | 0.037 | 37 ± 11 | 31 ± 13 | 0.004 | -3.68 ± 13.61 | -5.48 ± 14.15 | 0.477 |  |

RV, right ventricle

Values are mean ± SD. Differences between baseline and follow-up echocardiography, as well as the remodeling for RV end-systolic area and change in RV fractional area change were analyzed using the paired t-test.

**Table S4.** Percentage of reduction in RV end-systolic area and absolute change RV fractional area change, for the overall population and according to tertiles of inclusion (tertile 1: < 173days surgery – FU-TTE; tertile 2: 173 – 251 days surgery – FU-TTE; tertile 3: > 251days surgery – FU-TTE).

| **Remodeling per tertile** | **Total population**  **n = 121** | **Tertile 1**  **< 173 days**  **n = 41** | **Tertile 2**  **173 – 251 days**  **n = 41** | **Tertile 3**  **> 251 days**  **n = 39** | **p-value** |
| --- | --- | --- | --- | --- | --- |
| RV end-systolic area, cm^2^ | -4.95 ± 46.80 | 2.80 ± 32.62 | -13.50 ± 55.04 | -4.10 ± 49.62 | 0.288 |
| RV fractional area change, % | -4.56 ± 13.84 | -1.74 ± 14.78 | -7.65 ± 13.18 | -4.27 ± 13.18 | 0.152 |

RV, right ventricle.

Values are mean ± SD. Differences among the tertiles were analyzed using the one-way ANOVA test. Multiple comparisons for continuous variables were tested with the Bonferroni correction; none of the tertiles showed significant p-value <0.05 compared to one-another.

**Table S5.** Inter-observer reproducibility

|  | **Reader 1 (MCM)**  **n = 20** | **Reader 2 (XG)**  **n = 20** | **Mean difference**  **± SD** | **Error range** | **ICC (95%CI)** |
| --- | --- | --- | --- | --- | --- |
| RVEDA | 19 ± 5 | 20 ± 5 | -0.99 ± 3.26 | 4.52 | 0.874 (0.688 – 0.950) |
| RVESA | 12 ± 4 | 14 ± 4 | -1.57 ± 2.82 | 3.91 | 0.850 (0.575 – 0.943) |
| RVFAC | 38 ± 10 | 33 ± 11 | 5.08 ± 7.62 | 10.56 | 0.794 (0.379 – 0.924) |
| TAPSE | 13 ± 4 | 12 ± 4 | 1.64 ± 2.54 | 3.52 | 0.832 (0.485 – 0.939) |

ICC: intraclass correlation; RVEDA: right ventricular end-diastolic area; RVFAC: right ventricular fractional area change; RVESA: right ventricular end-systolic area; SD: standard deviation; TAPSE: tricuspid annular plane systolic excursion.

**Table S6.** Intra-observer reproducibility

|  | **1^st^ measurement**  **n = 20** | **2^nd^ measurement**  **n = 20** | **Mean difference**  **± SD** | **Error range** | **ICC (95%CI)** |
| --- | --- | --- | --- | --- | --- |
| RVEDA | 24 ± 8 | 25 ± 9 | -1.28 ± 3.43 | 4.75 | 0.952 (0.878 – 0.981) |
| RVESA | 18 ± 8 | 18 ± 7 | 0.20 ± 2.53 | 3.51 | 0.971 (0.926 – 0.988) |
| RVFAC | 27 ± 14 | 31 ± 11 | -4.02 ± 7.53 | 10.44 | 0.889 (0.683 – 0.958) |
| TAPSE | 11 ± 5 | 11 ± 4 | 0.15 ± 1.98 | 2.74 | 0.951 (0.877 – 0.981) |

ICC: intraclass correlation; RVEDA: right ventricular end-diastolic area; RVFAC: right ventricular fractional area change; RVESA: right ventricular end-systolic area; SD: standard deviation; TAPSE: tricuspid annular plane systolic excursion.

**Table S7.** Univariable Cox regression analysis for the absence of RV reverse remodeling.

| **Variable** | **RV end-systolic area** | | **RV fractional area change** | |
| --- | --- | --- | --- | --- |
|  | **Hazard ratio (95% CI)** | **p-value** | **Hazard ratio (95% CI)** | **p-value** |
| Age, years | 1.002 (0.976 – 1.028) | 0.890 | 0.988 (0.962 – 1.014) | 0.372 |
| Male gender | 1.189 (0.638 – 2.216) | 0.586 | 1.271 (0.664 – 2.432) | 0.469 |
| Arterial hypertension | 0.561 (0.301 – 1.044) | 0.068 | 0.579 (0.280 – 1.199) | 0.141 |
| Dyslipidemia | 1.260 (0.675 – 2.351) | 0.469 | 1.015 (0.501 – 2.060) | 0.966 |
| Diabetes mellitus | 1.201 (0.553 – 2.611) | 0.643 | 1.241 (0.563 – 2.732) | 0.592 |
| Smoking | 0.993 (0.515 – 1.917) | 0.984 | 1.073 (0.553 – 2.085) | 0.834 |
| Coronary artery disease | 1.076 (0.561 – 2.065) | 0.826 | 0.954 (0.485 – 1.879) | 0.892 |
| Atrial fibrillation | 1.215 (0.647 – 2.281) | 0.545 | 0.847 (0.446 – 1.607) | 0.611 |
| Pacemaker/ICD | 1.400 (0.730 – 2.683) | 0.311 | 1.099 (0.574 – 2.104) | 0.776 |
| Previous cardiac surgery | 1.079 (0.494 – 2.354) | 0.849 | 1.535 (0.736 – 3.199) | 0.253 |
| COPD | 0.922 (0.351 – 2.425) | 0.870 | 1.532 (0.616 – 3.807) | 0.358 |
| NYHA III or IV | 2.197 (1.094 – 4.412) | **0.027** | 0.939 (0.475 – 1.854) | 0.856 |
| Loop diuretic | 0.949 (0.463 – 1.947) | 0.887 | 0.804 (0.310 – 2.081) | 0.652 |
| Hemoglobin, mmol/L | 1.105 (0.874 – 1.397) | 0.402 | 0.970 (0.766 – 1.228) | 0.797 |
| Creatinine, µmol/L | 1.001 (0.996 – 1.007) | 0.671 | 0.999 (0.991 – 1.008) | 0.901 |
| Tricuspid valve annuloplasty | 21.859 (0.023 – 20618.583) | 0.377 | 4.738 (0.628 – 35.717) | 0.131 |
| Concomitant surgery | 1.412 (0.435 – 4.587) | 0.566 | 1.558 (0.602 – 4.036) | 0.361 |
| Left ventricular ejection fraction, % | 0.993 (0.973 – 1.013) | 0.490 | 0.981 (0.958 – 1.005) | 0.121 |
| LA end-systolic volume – indexed, mL/m^2^ | 1.002 (0.996 – 1.009) | 0.422 | 1.002 (0.997 – 1.007) | 0.450 |
| RV basal diameter, mm | 1.005 (0.975 – 1.036) | 0.749 | 0.989 (0.954 – 1.026) | 0.549 |
| RV mid diameter, mm | 0.991 (0.958 – 1.025) | 0.592 | 0.993 (0.960 – 1.028) | 0.703 |
| RV length, mm | 0.987 (0.964 – 1.011) | 0.282 | 1.002 (0.975 – 1.030) | 0.881 |
| RV end-diastolic area, mm^2^ | 0.960 (0.928 – 0.993) | **0.019** | 0.991 (0.957 – 1.026) | 0.612 |
| RV end-systolic area, mm^2^ | 0.939 (0.895 – 0.986) | **0.011** | 0.998 (0.953 – 1.046) | 0.939 |
| RV fractional area change, % | 1.016 (0.987 – 1.045) | 0.285 | 1.000 (0.968 – 1.033) | 0.992 |
| TAPSE, mm | 0.958 (0.896 – 1.023) | 0.199 | 1.000 (0.932 – 1.072) | 0.995 |
| RV peak systolic pressure, mmHg | 0.994 (0.976 – 1.013) | 0.528 | 1.015 (0.995 – 1.035) | 0.147 |
| Right atrial maximum area, mm^2^ | 1.003 (0.976 – 1.030) | 0.835 | 0.982 (0.955 – 1.010) | 0.202 |
| Tricuspid valve annular diameter, mm | 1.015 (0.979 – 1.052) | 0.412 | 0.972 (0.929 – 1.017) | 0.220 |

CI, confidence interval; ICD, implantable cardioverter-defibrillator; LA, left atrium; NYHA, New York Heart Association functional class; RV, right ventricle; and TAPSE, tricuspid annular plane systolic excursion.

Absence of right ventricular reverse remodeling was based on the relative percentage reduction in RV end-systolic area (corresponding to tertile 1: increase of at least 11.2% in RVESA) and absolute reduction in RV fractional area change (corresponding to tertile 1: increase of at least 11.7% in RVFAC).

**Table S8.** Multivariable Cox regression analysis for the absence of RV reverse remodeling.

| **Variable** | **Multivariable model 1** | | **Multivariable model 2** | |
| --- | --- | --- | --- | --- |
|  | **Hazard Ratio (95%CI)** | **p-value** | **Hazard Ratio (95%CI)** | **p-value** |
| Age, years |  |  | 1.005 (0.979 – 1.031) | 0.731 |
| Male gender |  |  | 1.312 (0.691 – 2.490) | 0.406 |
| NYHA III or IV | 2.724 (1.336 – 5.557) | 0.006 | 2.582 (1.260 – 5.292) | 0.010 |
| RV end-diastolic area, mm^2^ | 0.951 (0.918 – 0.985) | 0.006 | 0.948 (0.914 – 0.983) | 0.004 |

CI, confidence interval; NYHA, New York Heart Association functional class; and RV, right ventricle.

Absence of right ventricular reverse remodeling was based on the relative percentage reduction in RV end-systolic area (corresponding to tertile 1: increase of at least 11.2% in RVESA).
